# Development and economic assessment of machine learning models to predict glycosylated hemoglobin in type 2 diabetes

**DOI:** 10.3389/fphar.2023.1216182

**Published:** 2023-06-30

**Authors:** Yi-Tong Tong, Guang-Jie Gao, Huan Chang, Xing-Wei Wu, Meng-Ting Li

**Affiliations:** ^1^ Chengdu Second People’s Hospital, Chengdu, Sichuan, China; ^2^ Personalized Drug Therapy Key Laboratory of Sichuan Province, Department of Pharmacy, Sichuan Provincial People’s Hospital, School of Medicine, University of Electronic Science and Technology of China, Chengdu, Sichuan, China; ^3^ Chinese Academy of Sciences Sichuan Translational Medicine Research Hospital, Chengdu, Sichuan, China

**Keywords:** type 2 diabetes, glycosylated hemoglobin, prediction models, machine learning, model benefit

## Abstract

**Background:** Glycosylated hemoglobin (HbA1c) is recommended for diagnosing and monitoring type 2 diabetes. However, the monitoring frequency in real-world applications has not yet reached the recommended frequency in the guidelines. Developing machine learning models to screen patients with poor glycemic control in patients with T2D could optimize management and decrease medical service costs.

**Methods:** This study was carried out on patients with T2D who were examined for HbA1c at the Sichuan Provincial People’s Hospital from April 2018 to December 2019. Characteristics were extracted from interviews and electronic medical records. The data (excluded FBG or included FBG) were randomly divided into a training dataset and a test dataset with a radio of 8:2 after data pre-processing. Four imputing methods, four screening methods, and six machine learning algorithms were used to optimize data and develop models. Models were compared on the basis of predictive performance metrics, especially on the model benefit (MB, a confusion matrix combined with economic burden associated with therapeutic inertia). The contributions of features were interpreted using SHapley Additive exPlanation (SHAP). Finally, we validated the sample size on the best model.

**Results:** The study included 980 patients with T2D, of whom 513 (52.3%) were defined as positive (need to perform the HbA1c test). The results indicated that the model trained in the data (included FBG) presented better forecast performance than the models that excluded the FBG value. The best model used modified random forest as the imputation method, ElasticNet as the feature screening method, and the LightGBM algorithms and had the best performance. The MB, AUC, and AUPRC of the best model, among a total of 192 trained models, were 43475.750 (¥), 0.972, 0.944, and 0.974, respectively. The FBG values, previous HbA1c values, having a rational and reasonable diet, health status scores, type of manufacturers of metformin, interval of measurement, EQ-5D scores, occupational status, and age were the most significant contributors to the prediction model.

**Conclusion:** We found that MB could be an indicator to evaluate the model prediction performance. The proposed model performed well in identifying patients with T2D who need to undergo the HbA1c test and could help improve individualized T2D management.

## 1 Introduction

Diabetes mellitus (DM) is one of the most common and fastest-growing endocrine diseases, with both types (type 1 and type 2) contributing substantially to the healthcare costs of society. According to the International Diabetes Federation (IDF), the number of people with diabetes reached approximately 537 million worldwide by 2021 (1 in 10 adults live with diabetes), and approximately 90%–95% of cases of diabetes suffer from type 2 diabetes (T2D) (Sun et al., 2022). T2D has become a global threat to public health in the 21st century (Wang et al., 2022). Fasting blood glucose (FBG) and random blood glucose (RBG) have been the traditional method for assessing the risk of T2D, but they have obvious shortcomings—change over short periods of time due to behavioral changes (Christine et al., 2017). Relatively, glycated hemoglobin A1c (HbA1c), representing the average plasma glucose levels for the past 2–3 months (Rohlfing et al., 2002), has been recommended for diagnosing and monitoring diabetes by the World Health Organization (WHO) in 2011 and the American Diabetes Association (ADA) in 2010 (Leong et al., 2018).

According to the latest criteria, the American Diabetes Association (ADA) and the European Association for the Study of Diabetes (EASD) have recommended that glycemic management is evaluated primarily with the HbA1c test, and the therapeutic goal is to reduce the HbA1c to<7.0% (Davies et al., 2022). The Chinese guidelines are in line with international consensus—they stress the importance of regular HbA1c measurements (twice a year or four times a year) (Chinese Diabetes Society, 2021). Studies have shown that glycemic control is required in order to reduce the risk of onset and progression of complications (Williams et al., 2005; Yu et al., 2022). Once the target HbA1c is exceeded by 0.5% (>5 mmol/mol) after 3–6 months, further intensification should be administered. However, in practice, this does not always happen. The delay in intensifying therapy is referred to in clinical terms as therapeutic inertia and is due to underestimation of the need for therapy or failure to monitor the HbA1c level (Reach et al., 2017).

Machine learning (ML) is a branch of artificial intelligence and has been widely applied in clinical research and practice to construct high-performing prediction models, such as prediction of disease progression and outcomes (Griffith et al., 2020; Lewin-Epstein et al., 2020; Wang et al., 2020). Especially in the field of T2D management, identifying patients with T2D and estimating the risk of development of complications has become a hot topic during recent years (Ahlqvist et al., 2019; Dennis et al., 2019; Heerspink, 2019). ML has been shown to provide a useful management tool and has played a key role in the recognition of systems as routine therapeutic aids for patients with T2D. Thus, we consider whether it is possible to identify patients with a high risk of poor glycemic control utilizing machine learning methods based on the readily available daily data.

## 2 Methods

### 2.1 Data sources

The participants in the study were recruited from outpatients attending the Endocrinology Section of the Sichuan Provincial People’s Hospital. Participants were selected according to the following criteria: (1) over 18 years of age; (2) diagnosed as a T2D patient and received hypoglycemic treatment (the diagnostic criteria for T2D were in line with China’s 2017 guidelines on preventing and treating type 2 diabetes (Chinese Diabetes Society, 2017)); and (3) HbA1c levels were measured on the day of collection. (4) Researchers explained the purpose and scope of the survey to the subjects, and those who agreed to take part were retained in the study. Ethics approval was obtained through the Ethics Committee of the Sichuan Provincial People’s Hospital (approval # 2018-53).

Characteristics of participants were obtained from face-to-face interviews and electronic medical records (EMRs). The adherence status was defined according to the proportion of days covered (PDC). PDC higher than 80% was regarded as good medication compliance (Wu et al., 2020).

### 2.2 Outcome definition

HbA1c values on the day of visiting the clinic were measured at the clinical laboratory of Sichuan Provincial People’s Hospital and collected from EMRs. In this study, a value of HbA1c more than 7.0% was defined as positive and less than 7.0% was defined as negative. Furthermore, parents who had a positive HbA1c were considered to be needed for detection on the day of attending the Endocrinology Section.

### 2.3 Data pre-processing

After data collection ended, the information was converted to an Excel format. Each column represented a candidate variable, and each row represented a sample. To acquire high-quality data for modeling, a series of interventions were performed, including data pre-screening, data imputing, and variable selection.

First, data pre-screening was carried out using the following criteria: (1) the columns with missing values > 90% were removed, (2) the columns with a single value occupying >90% were removed, and (3) the columns with the coefficients of variation <0.1 were removed.

Missing information was inevitable in clinical data, such as the FBG value and PBG value. Missing data were filled using four imputing methods, including simple imputing (marked as SI), random forest imputing (marked as RF), k-nearest neighbor imputing (marked as KNN), and optimal deletion (marked as OD).

In order to eliminate irrelevant variables, reduce the number of variables, and improve the accuracy of the model, variable selection was performed. In this study, four algorithms—LASSO (Tibshirani, 1997), ridge regression (Marquardt and Snee, 1975), ElasticNet (Simon et al., 2011), and Boruta (Kursa M B, 2010)—were used to screen the key variables. The four aforementioned algorithms were marked as LA, RD, EN, and BOR, respectively.

### 2.4 Data partition

80 % of the data were assigned as the training set and the rest as the test set. The training set was used to train a classification model, and the test set was used to evaluate the model performance. Meanwhile, to assess whether the FGB value on the day was the important variable, the original data with the FGB value were used to train the models.

### 2.5 Model building

Sixteen datasets were generated in the training set by four data imputation methods and four variable selection methods. Then, six machine learning algorithms were employed on each dataset, respectively, to develop a total of 112 models. Machine learning algorithms in this study included random forest (RF), logistic regression (LR), multilayer perceptron (MLP), extreme gradient boosting (XGBoost), light gradient boosting machine (LGBM), and categorical boosting (CB).

RF, an ensemble learning algorithm proposed by Breiman, is very commonly used for classification (Breiman, 2001). Individual decision trees are built using a random subset of the training dataset in the training process. The final classification is then based on the majority voting results of all decision trees (Singha et al., 2019).

LR is widely used to solve binary classification problems (Jaillard et al., 2020). It predicts the probability of whether a dependent variable belongs to a particular class. The principle of LR is to first fit the decision boundary and then establish the probability relationship between the boundary and the classification so as to obtain the probability in the case of two classifications (Wang et al., 2020).

MLP, also known as a feed-forward neural network, is one of the most common deep learning approaches (Wan et al., 2018). It is mainly used to address supervised learning problems by learning the dependencies between the input layer (the variables) and output layer (the classification decision) using a fully connected hidden layer (Wang et al., 2020).

XGBoost (Chen and Guestrin, 2016), LightGBM (Guolin et al., 2017), and CatBoost (Bentéjac et al., 2021) were the three most popular implementations of gradient-boosting tree-based ensemble methods (Friedman, 2001). While built on structurally similar ideas, these libraries differ slightly on how decision trees are grown or how categorical variable data are handled, and only experimentation can validate which performs the best.

### 2.6 Model evaluation

The test set was used for external validation. A confusion matrix was used to evaluate the accuracy of classifier classification. In this study, a confusion matrix combined with relevant economic indicators, renamed as model benefit (MB), was used to redefine model performance to evaluate the accuracy of classifier classification. The test fee for HbA1c at our hospital was ¥73 per test. The mean additional economic burden of therapeutic inertia (Lindvig et al., 2021) was regarded as the cost of missed detection. In accordance with the current exchange rate, the exchange rate of the renminbi (RMB, ¥) against the Danish Krone is approximated to 1:1. The cost of missed detection was ¥786.77. The calculation formula was as follows:

$$MB\left(¥\right)=Total\;cost\left(TC,¥\right)-Model\;cost\left(MC,¥\right),$$

$$TC\left(¥\right)=Total\;participants\times HbA1c\;test\;fee,$$

$$MC\left(¥\right)=TP*\times HbA1c\;test\;fee+FP*\times HbA1c\;test\;fee+FN*\times cost\;of\;missed\;detection-TN*\times HbA1c\;test\;fee,$$

$$\left(*:TP=true\;positive,FP=false\;positive,FN=false\;negative,TN=true\;negative\right).$$

In addition, the area under the receiver operating characteristic curve (AUC), area under the precision recall curve (AUPRC), and decision curve analysis (DCA) were summarized to assess the model performance. The contribution of each variable to the predictive model was estimated with SHapley Additive exPlanation (SHAP).

### 2.7 Sample size validation

The best model (assessed by MB) was employed to estimate the impact of sample sizes on the predictive performance (Wu et al., 2020). The total samples were randomly separated into the training set and the test set at a ratio of 8:2. First, 10% of the training set was extracted to train the model, and the AUC was evaluated in the test set. The selected samples from the training set increased from 10% to 100% with a stepwise increase of 10%. These steps were repeated 10 times to generate 10 independent repeated AUC values. The relationship of sample size with the prediction performance of models was assessed according to the inflection point change of the line graph. The steeper broken line indicated that a larger sample size would improve the prediction performance of the model, and the gentler slope indicated that the performance of models was affected a little by the sample size.

**FIGURE 1:**
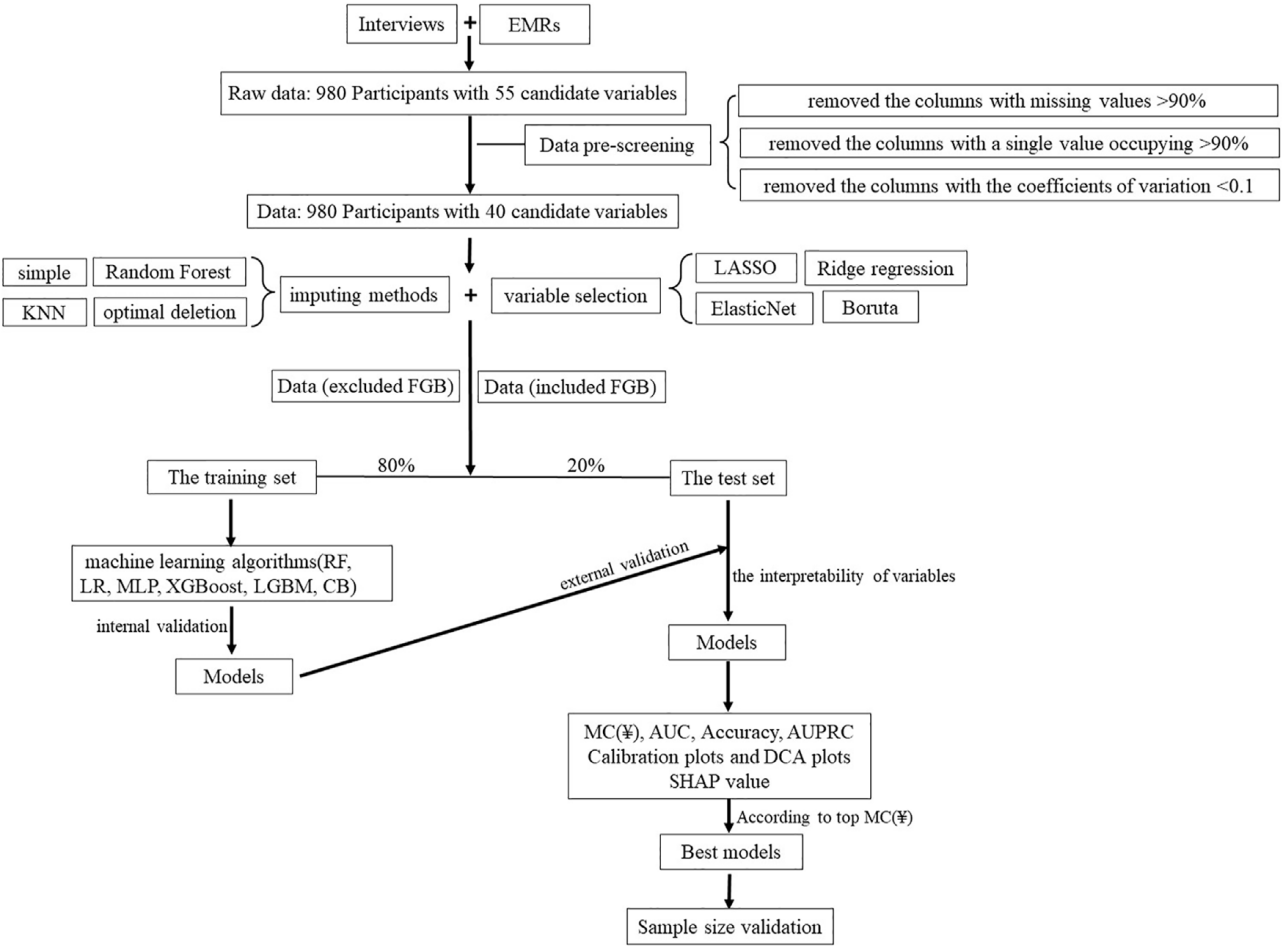
Overview of the main modeling steps.

Above all, the concise workflow for the development and validation of models is summarized in Figure 1.

### 2.8 Statistical analysis

The continuous variables were described by mean and standard deviation, whereas categorical variables were expressed in terms of frequencies and percentages. Multivariate analyses were performed to identify the factors associated with the model performance. Multivariate analysis was performed by multi-linear regression analysis. Model development was performed using the *sklearn* package and SHAP package in Python (*Python* Software Foundation, Python Language Reference, version 3.6.8) on PyCharm (developed by JetBrains.r.o., version 11.0.4). The grid search technique was applied to calculate hyperparameter values optimally.

## 3 Results

### 3.1 Participant characteristics

Overall, 980 patients completed the survey, among which 571 were male and 409 were female. The mean age was 59.2 ± 11.9 years. A total of 513 patients were defined as positive (52.3%). Participants were grouped according to the HbA1c value, and detailed characteristics of the participants are shown in Table 1.

**TABLE 1:** Characteristics of participants when grouped according to HbA1c.

Variable	Identifier	Parameter	Total	HbA1c ≤ 7.0	HbA1c > 7.0
HbA_1c_ values			980 (100%)	467 (47.7%)	513 (52.3%)
Age	X1		59.2 ± 11.9	58.3 ± 12.0	59.9 ± 11.8
Nationality	X2				
		Han	945 (96.5%)	456 (97.6%)	489 (95.3%)
		Tibetan	31 (3.2%)	9 (1.9%)	22 (4.3%)
		Qiang	3 (0.3%)	1 (0.2%)	2 (0.4%)
Gender	X3				
		Male	571 (58.3%)	267 (57.2%)	304 (59.2%)
		Female	409 (41.7%)	200 (42.8%)	209 (40.7%)
Waistline (cm)	X4		85.3 ± 9.5	85.0 ± 9.8	85.5 ± 9.3
BMI (kg/m^2^)	X5		24.3 ± 3.3	24.4 ± 3.6	24.18 ± 3.1
Marital status	X6				
		Unmarried	9 (0.9%)	7 (1.5%)	2 (0.4%)
		Married	940 (96.3%)	447 (95.7%)	493 (96.1%)
		Divorced	4 (0.4%)	1 (0.2%)	3 (0.6%)
		Widowed	23 (2.4%)	9 (1.9%)	14 (2.7%)
Occupational status	X7				
		Unemployed	133 (13.6%)	49 (10.5%)	84 (16.4%)
		Employed	358 (36.6%)	181 (38.8%)	177 (34.5%)
		Retirement	482 (49.3%)	234 (50.1%)	248 (48.3%)
		Others	5 (0.5%)	1 (0.2%)	4 (0.8%)
Education level	X8				
		Illiteracy	92 (9.4%)	31 (6.6%)	61 (11.9%)
		Junior middle school	366 (37.4%)	175 (37.5%)	191 (37.2%)
		High school or special secondary school	264 (27.0%)	125 (26.8%)	139 (27.1%)
		College and above educational level	256 (26.2%)	136 (29.1%)	120 (23.4%)
Family history of diabetes mellitus	X9				
		No	629 (64.8%)	304 (65.1%)	325 (63.4%)
		Yes	341 (35.2%)	157 (33.6%)	184 (35.9%)
Medicare status	X10				
		Non-reimbursement	233 (45.0%)	122 (26.1%)	111 (21.6%)
		Reimbursement	285 (55.0%)	132 (28.3%)	153 (29.8%)
Previous HbA_1c_ values (%)	X11				
		≤7%	269 (39.8%)	216 (46.3%)	53 (10.3%)
		7%–9%	328 (48.5%)	118 (25.3%)	210 (40.9%)
		>9%	79 (11.7%)	16 (3.4%)	63 (12.3%)
Interval of measurement (in days)	X12		212.5 ± 213.7	186.1 ± 168.9	243.5 ± 253.4
Course of diabetes (in months)	X13		90.3 ± 76.5	71.9 ± 68.0	107.0 ± 80.0
Frequency of FBG measurements	X14				
		Irregular measurements	139 (14.2%)	59 (12.6%)	80 (15.6%)
		Two to three times a week	323 (33.0%)	159 (34.0%)	164 (32.0%)
		Three to four times a month	400 (40.8%)	197 (42.2%)	203 (39.6%)
		Two to three times per 3 months	118 (12.0%)	52 (11.1%)	66 (12.87)
Duration of treatment regimen (in months)	X15		24.8 ± 34.0	21.7 ± 29.3	27.6 ± 37.5
Number of oral drugs	X16				
		0	71 (7.2%)	16 (3.4%)	55 (10.7%)
		1	328 (33.5%)	173 (37.0%)	155 (30.2%)
		2	419 (42.8%)	190 (40.7%)	229 (44.6%)
		3	153 (15.6%)	81 (17.3%)	72 (14.0%)
		4	8 (0.8%)	7 (1.5%)	1 (0.2%)
		5	1 (0.1%)	0	1 (0.2%)
Type of insulin used	X17				
		0	731 (74.6%)	383 (82.0%)	348 (67.8%)
		1	228 (23.3%)	80 (17.1%)	148 (28.9%)
		2	21 (2.1%)	4 (0.9%)	17 (3.3%)
Use of other types of drugs	X18				
		None	804 (82.1%)	392 (83.9%)	412 (80.3%)
		National medicine	11 (1.1%)	1 (0.2%)	10 (2.0%)
		Chinese medicine	88 (9.0%)	41 (8.8%)	47 (9.2%)
		Healthcare products	71 (7.3%)	31 (6.6%)	40 (7.8%)
		Others	5 (0.5%)	2 (0.4%)	3 (0.6%)
Type of operation or other communicable diseases	X19				
		No	775 (79.2%)	372 (80.0%)	403 (78.6%)
		Abdominal surgery	114 (11.6%)	48 (10.3%)	66 (12.9%)
		Thoracic surgery	31 (3.2%)	16 (3.4%)	15 (2.9%)
		Others	59 (6.0%)	31 (6.6%)	28 (5.5%)
Number of comorbid diseases	X20				
		0	500 (51.1%)	229 (49.0%)	271 (52.8%)
		1	299 (30.5%)	145 (31.0%)	154 (30.0%)
		2	143 (14.6%)	73 (15.6%)	70 (13.7%)
		3	34 (3.5%)	19 (4.1%)	15 (2.9%)
		4	3 (0.3%)	1 (0.2%)	2 (0.4%)
Hypertension	X21				
		No	663 (67.7%)	311 (66.6%)	352 (68.6%)
		Yes	317 (32.3%)	156 (33.4%)	161 (31.4%)
Hyperlipidemia	X22				
		No	768 (78.4%)	364 (77.9%)	404 (78.8%)
		Yes	211 (21.6%)	103 (22.1%)	108 (21.0%)
With or without complications	X23				
		No	884 (90.2%)	411 (88.0%)	473 (92.2%)
		Yes	96 (9.8%)	56 (12.0%)	40 (7.8%)
Vascular complications	X24				
		No	977 (99.7%)	465 (99.6%)	512 (99.8%)
		Yes	3 (0.3%)	2 (0.4%)	1 (0.2%)
Neurological complications	X25				
		No	926 (94.5%)	434 (92.9%)	492 (95.9%)
		Yes	54 (5.5%)	33 (7.1%)	21 (4.1%)
Complications with lesions of the extremities	X26				
		No	975 (99.5%)	466 (99.8%)	509 (99.2%)
		Yes	5 (0.5%)	1 (0.2%)	4 (0.8%)
Ocular complications	X27				
		No	973 (99.3%)	465 (99.6%)	508 (99.0%)
		Yes	7 (0.7%)	2 (0.4%)	5 (1.0%)
Nephropathy complications	X28				
		No	972 (99.2%)	461 (98.7%)	511 (99.6%)
		Yes	8 (0.8%)	6 (1.3%)	2 (0.4%)
Complications (other diseases)	X29				
		No	957 (97.7%)	453 (97.0%)	504 (98.3%)
		Yes	23 (2.3%)	14 (3.0%)	9 (1.7%)
Intensity of exercise	X30				
		None	153 (15.6%)	51 (10.9%)	102 (19.9%)
		Low intensity	664 (67.8%)	321 (68.7%)	343 (66.9%)
		Moderate intensity	124 (12.7%)	76 (16.3%)	48 (9.4%)
		High intensity	39 (3.9%)	19 (4.1%)	20 (3.9%)
Exercise session (mins/day)	X31		53.4 ± 55.4	53.5 ± 42.1	53.3 ± 65.3
Have a rational and reasonable diet	X32				
		No	256 (26.1%)	70 (15.0%)	186 (36.3%)
		Yes	724 (73.9%)	397 (85.0%)	327 (63.7%)
Sleep duration	X33				
		Good	453 (46.2%)	223 (47.8%)	230 (44.8%)
		Ordinary	333 (34.0%)	154 (33.0%)	179 (34.9%)
		Lose sleep	194 (19.8%)	90 (19.3%)	104 (20.3%)
Psychological status	X34				
		Well	459 (46.8%)	230 (49.3%)	229 (44.6%)
		General	493 (50.3%)	225 (48.2%)	268 (52.2%)
		Depression	28 (2.9%)	12 (2.5%)	16 (3.1%)
Health status scores (%)	X35		77.3 ± 10.8	78.3 ± 11.2	76.4 ± 10.3
EQ-5D scores	X36		0.9 ± 0.1	0.9 ± 0.1	0.9 ± 0.1
Medication adherence	X37				
		No	183 (18.6%)	47 (10.1%)	137 (26.7%)
		Yes	797 (83.4%)	420 (89.9%)	376 (73.3%)
Use of metformin	X38				
		None	313 (32.0%)	127 (27.2%)	186 (36.3%)
		Once a day	175 (17.9%)	97 (20.8%)	78 (15.2%)
		Twice a day	399 (40.8%)	198 (42.4%)	201 (39.2%)
		Three times a day	92 (9.3%)	44 (9.4%)	48 (9.4%)
Dose of metformin	X39				
		None	313 (32.1%)	127 (27.2%)	186 (36.3%)
		0.25 g	50 (5.1%)	18 (3.9%)	32 (6.2%)
		0.425 g	2 (0.2%)	2 (0.4%)	0
		0.5 g	154 (15.8%)	60 (12.9%)	94 (18.3%)
		0.75 g	1 (0.1%)	0	1 (0.2%)
		0.85 g	447 (45.8%)	256 (54.8%)	191 (37.2%)
		1.0 g	9 (0.9%)	2 (0.4%)	7 (1.4%)
Type of manufacturers of metformin	X40				
		Unknown	313 (32.1%)	127 (27.2%)	186 (36.3%)
		Generic drugs	205 (21.0%)	75 (16.1%)	130 (25.3%)
		Guthentic drugs	458 (46.9%)	264 (56.5%)	194 (37.8%)
α-Glucosidase inhibitors	X41				
		No	616 (62.9%)	290 (62.1%)	326 (63.5%)
		Yes	364 (37.1%)	177 (37.9%)	187 (36.5%)
Sulfonylureas	X42				
		No	637 (65.0%)	316 (67.7%)	321 (62.6%)
		Yes	343 (35.0%)	151 (32.3%)	192 (37.4%)
Glinides	X43				
		No	911 (93.0%)	439 (94.0%)	472 (92.0%)
		Yes	69 (7.0%)	28 (6.0%)	41 (8.0%)
DPP-4 inhibitors	X44				
		No	845 (86.2%)	379 (81.2%)	466 (90.8%)
		Yes	135 (13.8%)	88 (18.8%)	47 (9.2%)
Thiazolidinediones	X45				
		No	928 (94.7%)	438 (93.79%)	490 (95.52%)
		Yes	52 (5.3%)	29 (6.21%)	23 (4.48%)
GLP-1 RAs	X46				
		No	979 (99.9%)	466 (99.79%)	513 (100.00%)
		Yes	1 (0.1%)	1 (0.21%)	0
SGLT2 inhibitors	X47				
		No	976 (99.6%)	464 (99.36%)	512 (99.81%)
		Yes	4 (0.4%)	3 (0.64%)	1 (0.19%)
Use of Chinese medicine	X48				
		No	974 (99.4%)	467 (100.00%)	507 (98.83%)
		Yes	6 (0.6%)	0	6 (1.17%)
Use of insulin	X49				
		No	744 (75.9%)	385 (82.44%)	359 (69.98%)
		Yes	236 (24.1%)	82 (17.56%)	154 (30.02%)
Dose of non-basal insulin in the morning (U)	X50		2.2 ± 5.8	1.35 ± 4.76	2.95 ± 6.46
Dose of non-basal insulin in the noon (U)	X51		0.4 ± 2.5	0.25 ± 2.19	0.55 ± 2.76
Dose of non-basal insulin in the afternoon (U)	X52		2.2 ± 5.7	1.36 ± 4.67	2.93 ± 6.42
Dose of basal insulin (U)	X53		2.0 ± 5.7	1.25 ± 4.13	2.68 ± 6.78
Times of insulin use	X54				
		0	730 (74.5%)	381 (81.58%)	349 (68.03%)
		1	104 (10.6%)	45 (9.64%)	59 (11.50%)
		2	112 (11.4%)	33 (7.07%)	79 (15.40%)
		3	15 (1.5%)	5 (1.07%)	10 (1.95%)
		4	19 (2.0%)	3 (0.64%)	16 (3.12%)
Present FBG values (mmoL/L)	X55		9.3 ± 3.56	7.12 ± 1.45	10.73 ± 3.81

BMI: body mass index. HbA1c: glycated hemoglobin. FBG: fasting blood glucose. RBG: random blood glucose. EQ-5D: EuroQol five-dimension questionnaire. DPP-4 inhibitors: dipeptidylpeptidase-4 inhibitors. GLP-1 Ras: glucagon-like peptide-1 receptor agonists. SGLT2 inhibitors: sodium-dependent glucose transporters 2 inhibitors.

After data pre-screening, 15 variables were removed (nationality, marital status, with or without complications, vascular complications, neurological complications, complications with lesions of the extremities, ocular complications, nephropathy complications, complications (other diseases), glinides, thiazolidinediones, GLP-1 Ras, SGLT2 inhibitors, SGLT2 inhibitors, and the use of Chinese medicine).

### 3.2 Dataset building

A total of 60 datasets were set up by applying different imputing methods and variable selection methods with 41 variables. The different numbers of variables and samples in each dataset are listed in Table 2.

**TABLE 2:** Detailed information of 16 datasets.

Number	Imputing methods	Screening methods	Number of variables	Number of train samples
1	KNN	BOR	14	784
2	KNN	EN	33	784
3	KNN	LA	33	784
4	KNN	RD	16	784
5	OD	BOR	12	767
6	OD	EN	29	767
7	OD	LA	29	767
8	OD	RD	18	767
9	RF	BOR	13	784
10	RF	EN	33	784
11	RF	LA	33	784
12	RF	RD	17	784
13	SI	BOR	14	784
14	SI	EN	33	784
15	SI	LA	33	784
16	SI	RD	16	784

### 3.3 Model validation

**FIGURE 2:**
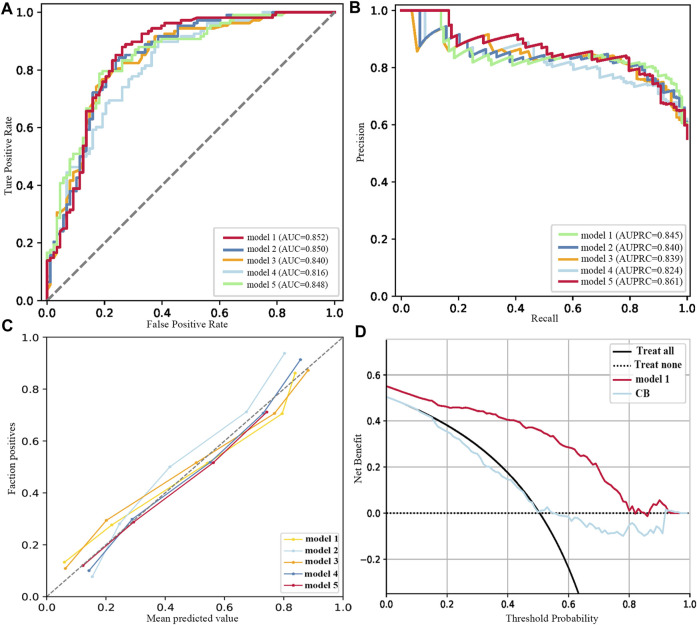
Performance of the five best models (excluded FBG). **(A)** ROC curves. **(B)** Precision–recall curves. **(C)** Calibration plots. **(D)** DCA plots.

A total of 192 models (whether they included FBG or not) were validated in the test set, considered as external validation, and the performance metrics were output. As shown in Table 3; Figure 2, the five best models (excluded FBG) were listed in sequence according to the MB value. The five best models were trained in the No. 12 dataset (applied modified random forest as the imputing method and Ridge as the selection method). The MB, AUC, accuracy, and AUPRC values of the best model (model 1) were 3163.800 (¥), 0.852, 0.811, and 0.845, respectively.

**TABLE 3:** Summary of the performance of the five best models (excluded the FBG value).

ID	Algorithms	No. of datasets	Parameters	MC(¥)	AUC	Accuracy	AUPRC
Model 1	LGBM	12	Max depth = 1	3163.800	0.852	0.811	0.845
			Subsample = 0.1				
Model 2	CB	12	N estimators = 96	−13950.450	0.850	0.791	0.840
Model 3	RF	12	N estimators = 197	−14031.600	0.840	0.770	0.839
Model 4	MLP	12	Activation = tanh; hidden layer sizes= (100, 50, 1); solver = sgd	−16221.600	0.816	0.755	0.824
Model 5	XGB	12	Gamma = 0.1; max depth = 3; subsample = 0.7	−18249.300	0.848	0.781	0.861

**FIGURE 3:**
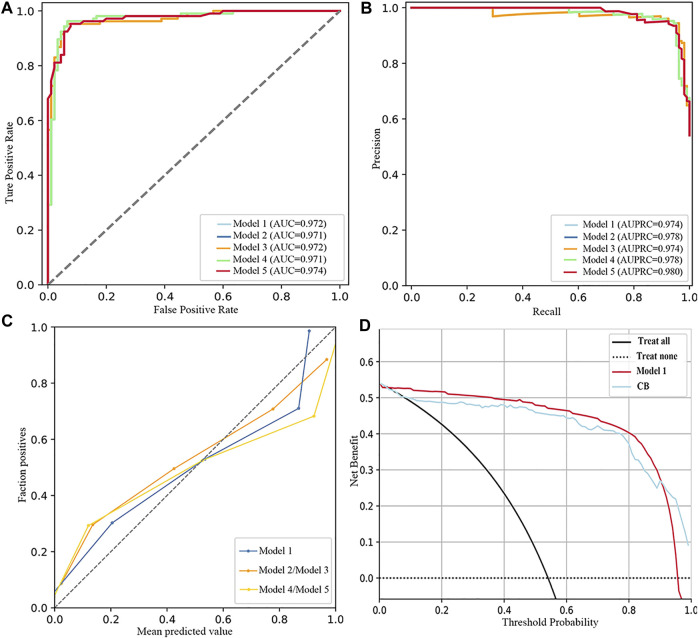
Performance of the five best models (included FBG). **(A)** ROC curves. **(B)** Precision–recall curves. **(C)** Calibration plots. **(D)** DCA plots.

As listed in Table 4; Figure 3, the five best models (included FBG) were trained in No. 11 and No. 10 datasets (applied modified random forest as the imputing method and ElasticNet or LASSO as the selection method). The MB, AUC, accuracy, and AUPRC values of the best model (model 1) were 43475.750 (¥), 0.972, 0.944, and 0.974, respectively. The calibration and DCA curves also displayed excellent predictive performances (Figures 2C, D; Figures 3C, D). The model that included FBG produced superior forecast performance compared to the model that excluded the FBG value.

**TABLE 4:** Summary of the performance of the five best models (included the FBG value).

ID	Algorithms	No. of datasets	Parameters	MC(¥)	AUC	Accuracy	AUPRC
Model 1	LGBM	10	Max depth = 5	43475.750	0.972	0.944	0.974
			Subsample = 0.1				
Model 2	CB	10	N estimators = 67	43475.750	0.971	0.944	0.978
Model 3	LGBM	11	Max depth = 5	43475.750	0.972	0.944	0.974
			Subsample = 0.1				
Model 4	CB	11	N estimators = 67	43475.750	0.971	0.944	0.978
Model 5	RF	10	N estimators = 147	42745.750	0.974	0.939	0.980

### 3.4 SHapley Additive exPlanation evaluation

**FIGURE 4:**
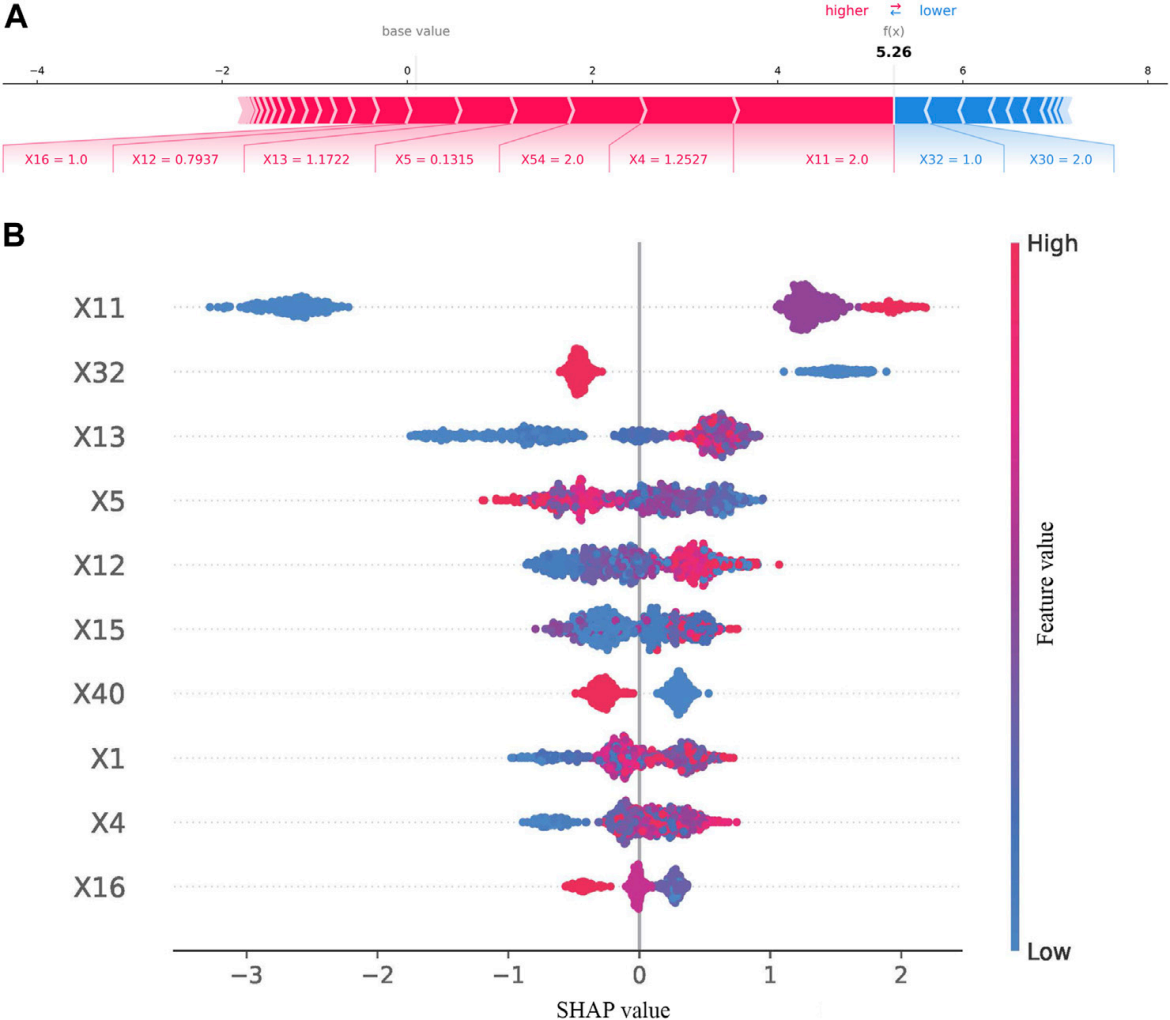
Results of SHAP in the best model (excluded FBG). **(A)** SHAP value contribution graph of each indicator of a single sample. **(B)** Complete distribution of the SHAP values for the top 10 variables.

SHAP was used to interpret the results from the best model. The result of SHAP in the best model (excluded FBG) is shown in Figure 4. As shown in Figure 4A, SHAP evaluation quantifies the contribution of a feature in a single sample. As results in Figure 4B, previous HbA1c values, having a rational and reasonable diet, course of diabetes, BMI, interval of measurement, duration of treatment regimen, type of manufacturers of metformin, age, waistline, and marital status were the 10 most important variables.

**FIGURE 5:**
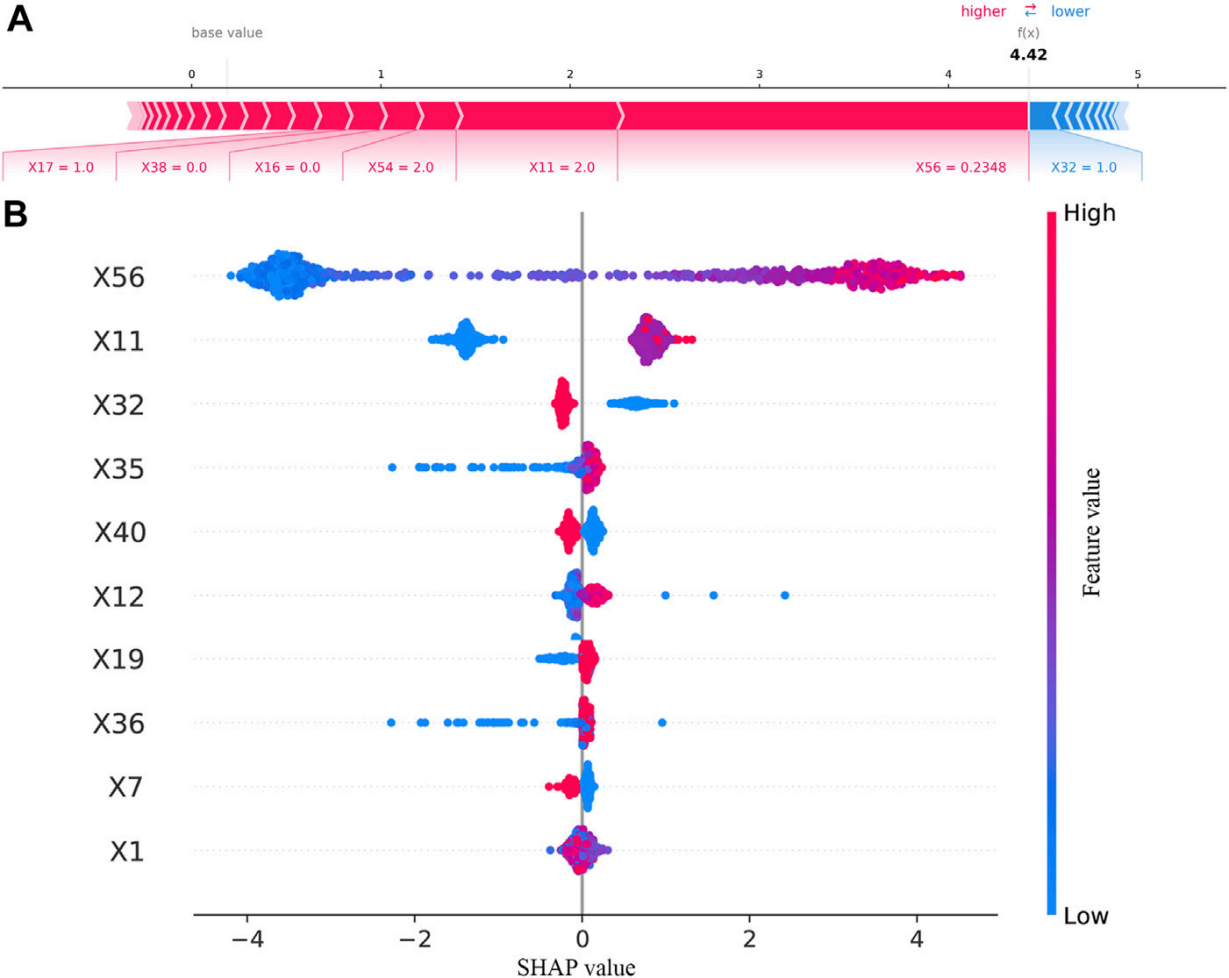
Results of SHAP in the best model (included FBG). **(A)** SHAP value contribution graph of each indicator of a single sample. **(B)** Complete distribution of the SHAP values for the top 10 variables.

The results of the contribution of variables in the best model (included FBG) are shown in Figure 5. As illustrated in Figure 5A, waistline, previous HbA1c values, interval of measurement, number of oral drugs, psychological status, EQ-5D scores, type of manufacturers of metformin, and FBG values provided a positive contribution to the SHAP value, while exercise session and course of diabetes provided a negative contribution. As presented in Figure 5B, the 10 most important variables were FBG values, previous HbA1c values, having a rational and reasonable diet, health status scores, type of manufacturers of metformin, interval of measurement, EQ-5D scores, occupational status, and age.

### 3.5 Sample size assessment

**FIGURE 6:**
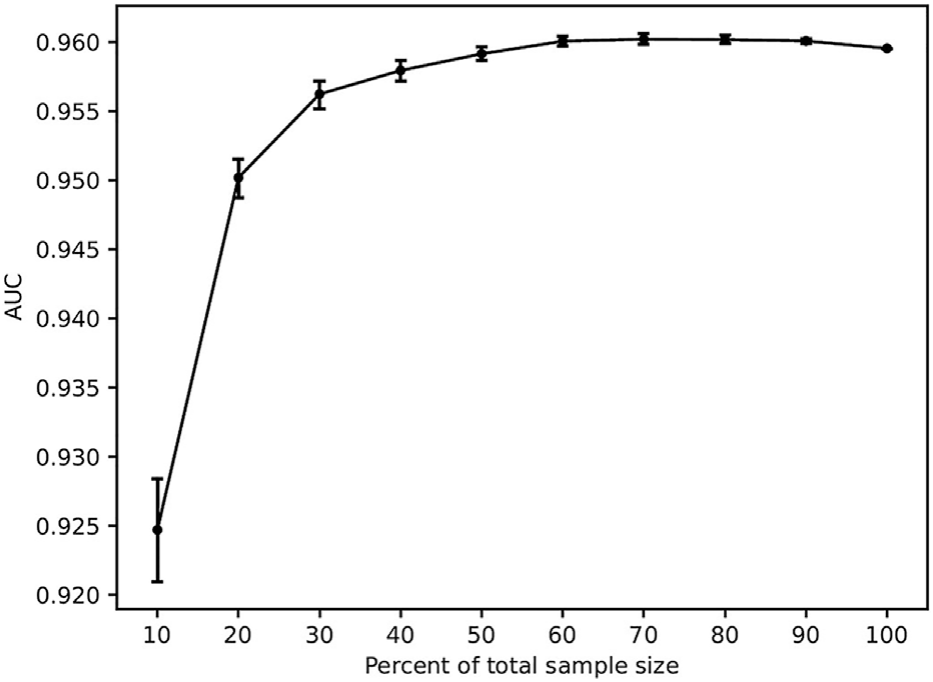
Results of sample size validation.

The adequacy of the sample size was verified using the resampling bootstrapping method, and the results are plotted in Figure 6. The AUC gradually increased and the dispersion of the AUC value decreased as the percentage of the sample size increased. When the sample size reached 60%, the curve flattened. The results indicated that the performance of the model might be slightly affected when expanding the sample size.

### 3.6 Multivariate analysis

As shown in Table 5, the number of samples could significantly affect the model prediction performance, including MB, AUC, accuracy, precision, and recall (*p* < 0.01). We found that the number of variables could affect the MB and recall of the prediction model significantly (*p* < 0.01). MB and AUC were influenced by screening methods (*p* < 0.05). Precision was affected by imputing methods (*p* < 0.01).

**TABLE 5:** Results of multivariate analysis.

Factors	MB(¥)	AUC	Accuracy	Precision	Recall
Number of samples	3149.651	−0.119	−0.02	−0.098	−0.015
	*p* < 0.001	*p* = 0.002	*p* < 0.001	*p* < 0.001	*p* < 0.001
Number of variables	−58.292	−0.001	-	-	−0.001
	*p* = 0.001	*p* = 0.817	-	-	*p* = 0.005
Imputing methods	−167.705	0.008	-	0.057	0.002
	*p* = 0.17	*p* = 0.610	-	*p* < 0.001	*p* = 0.196
Screening methods	−36.986	0.006		0.002	-
	*p* = 0.044	*p* = 0.011	-	*p* = 0.215	-

## 4 Discussion

In this research, we developed a total of 192 models (whether they included FBG or not) for the prediction of patients with poor glycemic control in patients with T2D. The MB, AUC, and AUPRC values of the best model were 43475.750 (¥), 0.972, 0.944, and 0.974, respectively. FBG values, previous HbA1c values, having a rational and reasonable diet, health status scores, type of manufacturers of metformin, interval of measurement, EQ-5D scores, occupational status, and age were the most important contributors to the prediction model.

In recent years, with the continuous development of artificial intelligence techniques, machine learning algorithms have been applied increasingly in clinical prediction models, and disease prediction models have begun to become a hot spot in clinical research. According to the TRIPOD checklist (Collins et al., 2015), the performance measures (with CI) for the prediction model should be reported. The AUC on validation data has represented the prediction abilities of models in most studies (Chan et al., 2021; Gibbons et al., 2021; de Souza et al., 2022; Yu et al., 2022). In addition, some prediction models have been internally validated by Harrell’s concordance index, the Brier score, and a satisfactory calibration curve (Qu et al., 2021; Lo-Ciganic et al., 2022). These aforementioned performance metrics pay more attention to the accuracy of the model and result in less clinical cost caused by wrong prediction or negative predictive value. In this study, we explored a novel measure that could overcome the limitation. Referring to the principles of pharmacoeconomic analysis, parameters for a cost–benefit analysis are costs for drugs and benefits for treatments. The worst outcomes of the absence of the HbA1c test were considered to lead to treatment inertia in this study. The economic burden associated with therapeutic inertia was regarded as the cost of negative predictive value, and these data were obtained from the study in patients with type 2 diabetes in Denmark (Lindvig et al., 2021). The fee for the HbA1c test was considered as the cost of treatment. Therefore, the MB of the best model in the study was 43475.750 (¥), suggesting that significant gains may result from the prediction model.

The primary goals in the treatment of patients with T2D are to maintain blood glucose levels as close to normal as possible and to achieve a relatively normal quality of life. Scientists early realized that both of these goals are influenced by a multitude of somatic and psychological factors (Rose et al., 2002; Williams et al., 2005). In addition, studies reported that educational level, age, duration of diabetes, BMI, and HbA1c at baseline were associated with HbA1c (Hu et al., 2020). One research reminded that occupational categories were relational to T2D (Baek et al., 2019). According to the results of SHAP in our study, the 10 most important variables were FBG values, previous HbA1c values, having a rational and reasonable diet, health status scores, type of manufacturers of metformin, interval of measurement, EQ-5D scores, occupational status, and age. The relationship between HbA1c and average glucose levels has been explored in many studies (Law et al., 2017). Meanwhile, this study developed prediction models on the different data (excluded FGB vs. included FGB). The results suggested that incorporating FGB into the models can allow for further improvements in predictive performance (3163.800 (¥) vs. 43475.750 (¥)).

In this study, multiple methods and algorithms were applied to build models. Because of their different principles, the methods and algorithms have different strengths and weaknesses. Specifically, four imputing methods were used to fill in missing values. The SI method fills with fixed values (Löw et al., 2019): the missing value of a continuous variable is replaced by the mean of the variable, and the missing value of a categorical variable is filled with the median. KNN (Beretta and Santaniello, 2016) and RF (Liao et al., 2022) are ensemble prediction methods and put out the predictive value to fill in the missing value of variables based on the variables without missing value. Compared to the fixed value, the predicted value should theoretically be similar to the true value. Meanwhile, this will also artificially increase the connection between variables. OD is a normal method to exclude variables with missing values, which we recently proposed. The principle of the algorithm was to keep the maximum sample size with no missing value by deleting variables (columns of the table) or samples (rows of the table). According to the results of the multivariate analysis (shown in Table 5), methods and algorithms could significantly affect the prediction performance. So, it is necessary to try which method is the most suitable for data preprocessing or modeling. On the same lines, XGBoost, LGBM, and CatBoost were implementations of gradient-boosting tree-based ensemble methods. The MB of LGBM was higher than that of others both in data that excluded the FGB value or data that included the FGB value (Table 3; Table 4), which was similar to a previous research (Zhang et al., 2022).

## 5 Limitation

First, the data were collected prospectively, but our study has the inherent limitations of a single-center retrospective analysis. Although the sample size in our study has been demonstrated to be suitable for modeling, more samples need to be collected in order to verify this prediction model, or a large multicenter sample study is desired that can substantiate the applicability of the model. Second, due to the retrospective research, for some variables, recall bias still exists, such as the intensity of exercise and exercise sessions.

## 6 Conclusion

In summary, the present research introduced 192 machine learning models to predict poor glycemic control in patients with T2D and proposed a new indicator to evaluate the performance of the prediction model. In fact, we developed a prediction model with better classifier performance. This work also reconfirmed that variables such as FBG values, previous HbA1c values, having a rational and reasonable diet, health status scores, interval of measurement, EQ-5D scores, occupational status, and age were risk factors for glycemic control. We are in the process of developing a mobile app or a Web server for caregivers and patients in an effort to integrate the glycemic control enhancement intervention into daily T2D management.

## Data Availability

The original contributions presented in the study are included in the article/Supplementary Material; further inquiries can be directed to the corresponding authors.
